# CAMP@FLASH: an end-station for imaging, electron- and ion-spectroscopy, and pump–probe experiments at the FLASH free-electron laser

**DOI:** 10.1107/S1600577518008585

**Published:** 2018-08-02

**Authors:** Benjamin Erk, Jan P. Müller, Cédric Bomme, Rebecca Boll, Günter Brenner, Henry N. Chapman, Jonathan Correa, Stefan Düsterer, Siarhei Dziarzhytski, Stefan Eisebitt, Heinz Graafsma, Sören Grunewald, Lars Gumprecht, Robert Hartmann, Günter Hauser, Barbara Keitel, Clemens von Korff Schmising, Marion Kuhlmann, Bastian Manschwetus, Laurent Mercadier, Erland Müller, Christopher Passow, Elke Plönjes, Daniel Ramm, Dimitrios Rompotis, Artem Rudenko, Daniela Rupp, Mario Sauppe, Frank Siewert, Dieter Schlosser, Lothar Strüder, Angad Swiderski, Simone Techert, Kai Tiedtke, Thomas Tilp, Rolf Treusch, Ilme Schlichting, Joachim Ullrich, Robert Moshammer, Thomas Möller, Daniel Rolles

**Affiliations:** a Deutsches Elektronen-Synchrotron (DESY), Hamburg, Germany; b Technische Universität Berlin, Berlin, Germany; c Center for Free-Electron Laser Science (CFEL), DESY, Hamburg, Germany; d Department of Physics, University of Hamburg, Hamburg, Germany; e Max Born Institute, Berlin, Germany; f PNSensor GmbH, Munich, Germany; g Max-Planck-Institut für Extraterrestrische Physik, Garching, Germany; h Max Planck Institute for Structure and Dynamics of Matter, Hamburg, Germany; i J. R. Macdonald Laboratory, Department of Physics, Kansas State University, Manhattan, KS, USA; j Helmholtz Zentrum Berlin für Materialien und Energie, Berlin, Germany; k Universität Siegen, Siegen, Germany; l Max Planck Institute for Biophysical Chemistry, Göttingen, Germany; m Institute for X-ray Physics, Göttingen University, Göttingen, Germany; n Max-Planck-Institut für Medizinische Forschung, Heidelberg, Germany; o Physikalisch-Technische Bundesanstalt, Braunschweig, Germany; p Max-Planck-Institut für Kernphysik, Heidelberg, Germany

**Keywords:** free-electron laser, soft X-rays, pump–probe, electron-ion-spectrometers, imaging detector, micro-focus, Kirkpatrick–Baez mirrors

## Abstract

The non-monochromatic beamline BL1 at the FLASH free-electron laser facility at DESY was upgraded with new transport and focusing optics, and a new permanent end-station, CAMP, was installed. This multi-purpose instrument is optimized for electron- and ion-spectroscopy, imaging and pump–probe experiments at free-electron lasers. It can be equipped with various electron- and ion-spectrometers, along with large-area single-photon-counting pnCCD X-ray detectors, thus enabling a wide range of experiments from atomic, molecular, and cluster physics to material and energy science, chemistry and biology. Here, an overview of the layout, the beam transport and focusing capabilities, and the experimental possibilities of this new end-station are presented, as well as results from its commissioning.

## Abstract 2

Beamline BL1 at the FLASH free-electron laser facility at DESY was upgraded with new transport and focusing optics for the installation of the new permanent CAMP end-station, a multi-purpose instrument optimized for electron- and ion-spectroscopy, imaging and pump–probe experiments. An overview of the layout, beam transport, focusing capabilities, and experimental possibilities of this new end-station, as well as results from its commissioning and first experiments, are presented.

## 1. Introduction   

Free-electron lasers (FELs) deliver coherent extreme ultraviolet (XUV) and X-ray pulses with unprecedentedly high intensities (>10^12^ photons/pulse) and ultra-short pulse durations (3–300 fs) (McNeil & Thompson, 2010 ▸). This opens the door towards numerous applications in a wide range of fields from atomic, molecular and optical physics to material and energy science, chemistry and biology, allowing, for example, single-shot coherent diffractive imaging, femto­second pump–probe and multi-photon ionization experiments in the XUV and X-ray domain. In particular, this enables a variety of novel imaging schemes to study ultra-fast processes with down to Ångström spatial and femtosecond temporal resolution. To address these new possibilities, the CFEL-ASG Multi-Purpose instrument (CAMP), optimized for imaging and pump–probe experiments, was developed in the Max-Planck Advanced Study Group (ASG) at the Center for Free-Electron Laser Science (CFEL) in Hamburg (Strüder *et al.*, 2010 ▸). Numerous pioneering experiments made use of the instrument at the AMO beamline of the Linac Coherent Light Source (LCLS) (Emma *et al.*, 2010 ▸) between 2009 and 2012 (Chapman *et al.*, 2011 ▸; Seibert *et al.*, 2011 ▸; Hau-Riege *et al.*, 2012 ▸; Gorkhover *et al.*, 2012 ▸; Aquila *et al.*, 2012 ▸; Barty *et al.*, 2011 ▸; Loh *et al.*, 2012 ▸; Starodub *et al.*, 2012 ▸; Rudek *et al.*, 2012 ▸, 2013 ▸; Boll *et al.*, 2013 ▸, 2014 ▸; Erk *et al.*, 2013*a* ▸,*b* ▸, 2014 ▸; Rolles *et al.*, 2014 ▸; Küpper *et al.*, 2013 ▸; Hantke *et al.*, 2014 ▸; Gomez *et al.*, 2014 ▸; van der Schot *et al.*, 2015 ▸; Ekeberg *et al.*, 2015 ▸; Tanyag *et al.*, 2015 ▸; Gorkhover *et al.*, 2016 ▸; Jones *et al.*, 2016 ▸). In 2014, CAMP was installed at the Free-electron LASer in Hamburg (FLASH) (Ackermann *et al.*, 2007 ▸; Faatz *et al.*, 2016 ▸), at beamline BL1 in the FLASH1 experimental hall as a permanent end-station for user experiments.

The CAMP end-station’s modular and flexible layout allows for simultaneous use of large-area single-photon-counting pnCCD photon-detectors and VMI- or COLTRIMS-type electron- and ion-spectrometers, with various (user provided) solid-state sample holders, gas beams, cluster sources, liquid-jets, (nano-)particle injectors and other sample delivery systems. For experiments using optical pulses as pump or probe, the end-station has dedicated laser in-coupling optics and diagnostics provided in a permanent laser setup at the BL1 beamline for use with the FLASH1 pump–probe lasers (Redlin *et al.*, 2011 ▸). In addition, the beamline is equipped with a dedicated XUV split-and-delay unit (DESC) (Sauppe *et al.*, 2018 ▸) that is based on multilayer mirrors and can provide two pulses with variable delay ranging from (sub-)femto­second to 650 ps.

In this paper, we present an overview of the layout and capabilities of the beamline including new Kirkpatrick–Baez (KB) focusing optics (Kirkpatrick & Baez, 1948 ▸) as well as the permanent end-station, CAMP@FLASH, by showing first results from its commissioning.

## 2. BL1 beamline and CAMP end-station layout   

### 2.1. Beamline layout and transmission   

The transport and focusing optics of beamline BL1 were upgraded to provide a tightly focused FEL beam over the entire wavelength range available at FLASH1, *i.e.* from 51 nm to 4.2 nm. In particular, the former toroidal focusing mirror at BL1 (BL1M1) providing a 100 µm focus was replaced by a flat transport mirror and the beam is now focused by a pair of KB mirrors (Kirkpatrick & Baez, 1948 ▸), installed within a BMBF collaborative research project, to provide micro-focusing capabilities (see §2.2).

**Figure 1:**
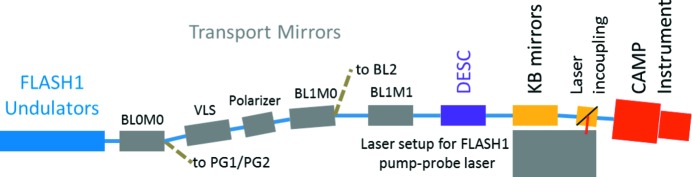
Sketch of the beamline transport and focusing mirrors from the undulators to the CAMP end-station. The FEL beam passes a total of six optics from the undulators towards the end-station. Additionally, behind the three flat transport mirrors BL0M0, BL1M0 and BL1M1, and the VLS, the optional multilayer-based split-and-delay unit (DESC) can be inserted into the FEL beam to create two XUV pulses for pump–probe experiments. The FEL beam is focused into the CAMP end-station by a set of two plane-elliptical Kirkpatrick–Baez (KB) mirrors with focal lengths of 810 mm (horizontal focusing) and 550 mm (vertical focusing), respectively.

**Figure 2:**
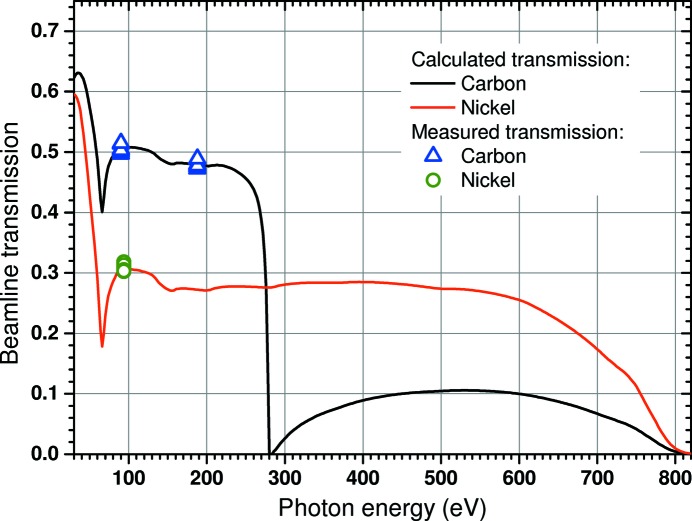
Total beamline transmission between the gas monitor detector placed behind the undulators in the beam-distribution area (BDA) and the CAMP interaction region. Solid curves are calculated values for both options of transport-mirrors. Colored symbols show measured transmissions at different photon energies using two 3 mm-diameter apertures in front of all the beamline optics, to prevent geometrical beam transmission loss in the transport and focusing mirrors. (Individual errors are smaller then the symbol size.)

The new beamline layout, including transport and focusing mirrors, an XUV split-and-delay unit and the CAMP end-station, is sketched in Fig. 1 ▸. The FEL beam passes a total of six optical elements from the undulators towards the end-station. There are three flat mirrors that have a two-area coating of carbon and nickel, which can be chosen according to the FEL wavelength. Respective transmission values are given in Fig. 2 ▸. One out of three variable-line-spacing (VLS) gratings can optionally be used to measure single-shot spectra in parallel to the experiment (Brenner *et al.*, 2011 ▸) or the beam can be reflected by an either nickel- or carbon-coated flat mirror. The beam is focused by two plane-elliptical mirrors, which will be discussed in detail in §2.2. More details on the FLASH1 beamlines, including BL1, are given by Tiedtke *et al.* (2009 ▸) and Feldhaus (2010 ▸).

Between the VLS and BL1M0, a device for polarization control (von Korff Schmising *et al.*, 2017 ▸) of the FEL beam, available for all BL beamlines, is now available to users. The polarizer is based on phase retardation upon reflection off metallic mirrors. It enables efficient and broadband generation of circularly polarized radiation in the extreme ultraviolet spectral range from 35 eV to 90 eV, with a degree of circular polarization reaching up to 90% while maintaining high total transmission values exceeding 30%.

For time-resolved XUV-pump XUV-probe experiments, the FEL beam can be geometrically split into two parts with a controllable splitting ratio and time delay by a multilayer-based setup called DElay-Stage for Camp (DESC), which can be inserted in the FEL beam path on demand. Due to the multilayer-mirror-based design, DESC can provide two FEL beams of the same photon energy with a very large delay range from −29 ps to +654 ps. The full delay range can be controlled with 200 as (mechanical) accuracy, due to a combination of both stepper-motor and piezo-crystal-driven in-vacuum delay-stages. The two beams overlap in the focus of the KB mirror system for XUV-pump XUV-probe experiments; more details of this setup are given by Sauppe *et al.* (2018 ▸).

The total transmission of the beamline optics, including the KB focusing mirrors, is shown in Fig. 2 ▸. Solid lines depict the calculated total transmission for two configurations, where the transport mirrors and the VLS optics are either aligned to the carbon- or nickel-coated areas of the mirrors, respectively. All transport mirrors are flat with 2° grazing-incidence angles with an effective area, *i.e.* the area of the mirror surface that is high-precision polished and coated, of 20 mm (vertical) by 500 mm (horizontal), respectively. The KB-optics have 3° grazing-incidence angles, a polished nickel-coated area of 10 mm by 195 mm, resulting in an effective area of about 10 mm by 10 mm, transverse to the beam propagation. This also results in the beam exiting the KB system to point 6° downwards and sideways which also results in a 6° downwards tilt of the whole experimental apparatus.

The transmission curves shown in Fig. 2 ▸ were calculated using tabulated theoretical reflectivity values (Henke *et al.*, 1993 ▸). The measured values of the total transmission are represented by symbols. To measure these values, we compared the calibrated gas monitor detector (GMD) (Tiedtke *et al.*, 2008 ▸) signal in front of mirror BL0M0 with the signal of another, mobile, GMD directly behind the CAMP end-station. Each data point represents an independent measurement taken at different times during 24 h of self-amplified spontaneous emission operation. All measurements shown in Fig. 2 ▸ were taken with a set of two 3 mm-diameter apertures in front of the beamline optics. This confines the beam divergence in a way that the transmitted beam is smaller than the active surface of the mirrors and thus the transmission (after the apertures) is not affected by the geometrical mirror acceptance. For longer wavelengths (>13.5 nm), the beam divergence can increase and the beam size becomes limited by the size of the polished coated mirror surfaces and the mechanical free path. For a set of two 3 mm-diameter apertures, which limit the beam size in a controlled way and prevent clipping the beam on the KB-mirrors, and therefore reduce stray light, the calculated and measured transmissions agree very well.

The nickel coating of the KB-mirrors, together with the dual-coating of the beamline transport mirrors, allows for strongly focusing the XUV beam over the whole energy range available from the FLASH1 undulators, including photon energies above the carbon *K*-edge. The overall transmission (not taking into account geometrical mirror acceptances, relevant below 100 eV) is about 48% below 250 eV and about 28% between 250 eV and 600 eV (for use with higher harmonics of FLASH).

### 2.2. Micro-focusing KB optics   

Before entering the CAMP end-station, the FEL beam is focused by a set of two nickel-coated plane-elliptical KB mirrors. The focal lengths of the KB mirrors are 810 mm (horizontal focus, first mirror) and 550 mm (vertical focus, second mirror), respectively.

The focusing optics and mechanics (Noll, 2004 ▸) were developed and manufactured in a collaboration between Technical University Berlin (T. Möller group), DESY, HZB (NOM team, F. Siewert and Th. Zeschke) as well as Carl Zeiss Optics.

**Figure 3:**
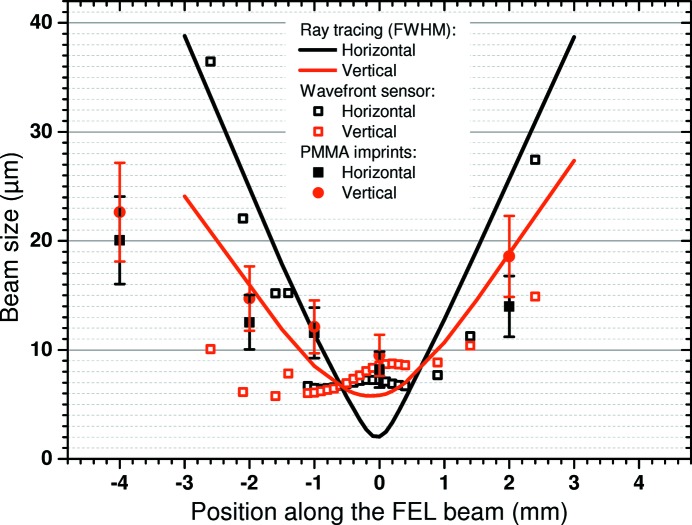
Results of the imprint measurements of the focused FLASH beam (13.5 nm, 100 fs) around the interaction point inside the CAMP end-station. The beam was attenuated to different fractions of the initial fluence using a set of filters for each longitudinal position of the PMMA-coated silicon wafer (PMMA thickness 2.5 µm). Solid symbols show beam sizes measured by PMMA imprints, empty symbols show wavefront sensor measurements, and solid lines show the results of ray-tracing calculations. The measured Rayleigh length, *i.e.* the path along the beam where the beam waist is smaller than 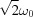, is about 1 mm, compared with <0.5 mm in the ray-tracing calculations.

The design values for the focus size of the mirror combination were 2 µm (H) by 3 µm (V) (FWHM) for slope errors of 0.1 µrad (r.m.s.). During the manufacturing process of the mirrors, the quality of the active mirror surface has been checked using the Nanometre Optical Component Measuring Machine (NOM) at the BESSY-II Optics Laboratory of the Helmholtz Zentrum Berlin (Siewert *et al.*, 2011 ▸) in two iterations. The mirrors were characterized by slope-measuring deflectometry before and after deterministic surface finishing by means of ion beam figuring (IBF) at Carl Zeiss (Thiess *et al.*, 2010 ▸). Using the three-dimensional slope mapping data, the topography of the mirrors was obtained and, based on this set of data, the dwell time-driven removal rate for the IBF-process was calculated. Ray-tracing simulations using the software *RAY* (Schäfers, 2008 ▸; Baumgärtel *et al.*, 2016 ▸), taking into account the full measured surface profiles, allowed assessment of the resulting focus quality before the integration of the mirrors. The final measured slope errors of 0.23 µrad (horizontal focus) and 0.13 µrad (vertical focus) led to a ray-traced beam waist of about 2 µm by 6 µm, as shown in Fig. 3 ▸. A source diameter of 235 µm (FWHM) and a beam divergence of 40 µrad (r.m.s.) were assumed (typical parameters for FLASH at wavelengths of 13.5 nm).

The mirror combination has been aligned by a series of iterative optimizations using wavefront-sensor measurements (Keitel *et al.*, 2016 ▸), and has been cross-checked using xenon time-of-flight spectra. At the optimized KB-mirror settings, the wavefront propagation calculations, based on the measurements with the wavefront sensor, yield a spot size of 6.1 µm by 6.5 µm (FWHM). In addition, a series of imprints (Liu, 1982 ▸; Chalupský *et al.*, 2010 ▸) on 2.5 µm PMMA (poly methyl methacrylate) coated silicon wafers (Silson UK) at four different fluences for each longitudinal position was performed and measured with a dedicated diagnostics chamber (Gerasimova *et al.*, 2013 ▸) using the same mirror alignment. The imprint measurements show a spot size of about 8 µm (H) by 9 µm (V) (FWHM) in the center of the CAMP chamber. Fig. 3 ▸ shows the results of the imprint measurements (13.5 nm, 100 fs pulses), the wavefront propagation calculations, as well as the ray-tracing calculations. The Rayleigh length, *i.e.* the path along the beam where the beam waist is smaller than 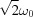, is about 1 mm in our measurements as compared with less than 0.5 mm in the ray-tracing calculations. This can be explained by remaining astigmatism in the KB-focusing that is in fact observed in both the WFS and imprint measurements in Fig. 3 ▸. The relative position along the beam of the three independent measurements and calculations shown in Fig. 3 ▸ with respect to each other has been placed to the best of our knowledge, but includes a relatively large uncertainty of the order of 1 mm.

We estimate the focus size to be in between these two values, as the imprint measurements, at the fluences used here, tend to overestimate the focus size, while the wavefront propagation may underestimate the focus size as the calculation assumes full transverse coherence. It is also important to consider that, for different wavelengths and other FEL parameters, the source size, source position along the undulators, and FEL beam divergence change, which affects the focus size and focus position.

To explore the capabilities of the strongly focused XUV beam, we used an ion time-of-flight spectrometer to record the yields of xenon charge states produced by (multiple) 4*d*-shell photo-ionization and subsequent Auger decay. Xenon gas has been introduced into the main chamber by means of a needle dosing-valve reaching a pressure of 2 × 10^−7^ mbar, while the base chamber pressure was at 1 × 10^−8^ mbar. In order to limit the ion-acceptance of the spectrometer along the beam to the focal volume, we used a 1 mm-wide slit-aperture on the extractor electrode with the slit being oriented perpendicular to the FEL beam propagation direction. For this measurement, the FEL parameters were 60 µJ single pulse energy on target (∼120 µJ at BDA-GMD), 90 fs pulse length (FWHM) and 92 eV photon energy, resulting in a focused intensity of 1 × 10^15^ W cm^−2^, assuming a 7 µm by 8 µm focus spot (based on the above estimate).

**Figure 4:**
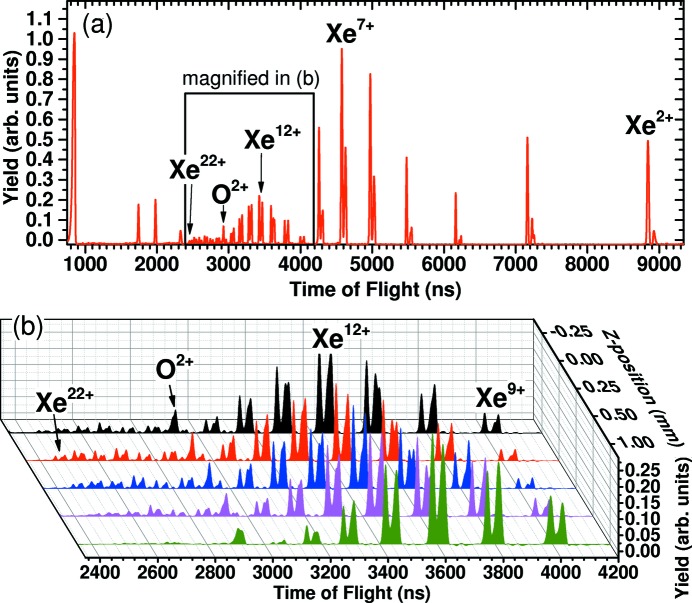
Using an FEL photon energy of 92 eV and a pulse energy of ∼60 µJ (120 µJ at BDA-GMD), 90 fs pulse length and 92 eV photon energy, charge states of xenon up to 22+ have been observed in the focus. (*a*) Xenon time-of-flight spectrum measured in the focus of the CAMP end-station (*z* = 0.00mm). (*b*) Moving the spectrometer, with a limited acceptance, along the FEL beam in the range −0.25 mm to +1.00 mm shows significant changes in the yields of high charge states of xenon, demonstrating the strong divergence of the beam and its effect on non-linear intensity-dependent effects.

The resulting xenon time-of-flight spectra are shown in Fig. 4 ▸. Moving the TOF spectrometer along the beam propagation axis, we aligned the spectrometer slit-aperture to the focal volume by maximizing the yield of the highest charge states. This method was found to be sensitive to movements of 0.1 mm along the beam propagation direction, showing significant increase in yields for xenon ion charge states above 16+.

The maximum charge state of xenon ions that was observed in these measurements was 22+, which is comparable with other measurements performed at FLASH (Sorokin *et al.*, 2007 ▸) where xenon 21+ was observed using a multilayer back-reflecting mirror tailored for a specific wavelength.

### 2.3. Layout of the CAMP end-station   

**Figure 5:**
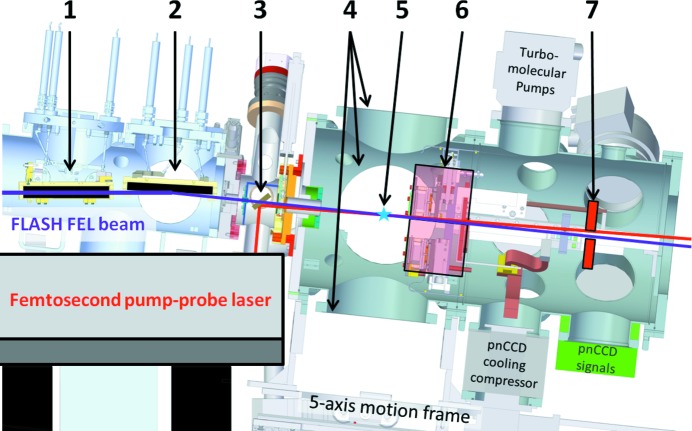
Layout of the CAMP end-station at FLASH BL1. The FEL beam enters from the left and is focused by the (1) horizontal and (2) vertical plane-elliptical focusing KB-mirrors. (3) The differential pumping section houses a drilled 1.5-inch mirror that is used to couple in the FLASH1 pump–probe laser and to overlap it with the FEL beam under a small angle. (4) The main experimental chamber has four DN250CF ports each in a transverse distance of 260 mm to (5) the interaction region, where the FEL is focused. Typically, the spectrometers (see §3.2) are inserted from the top and bottom, while sample injection or target holders are mounted on the sides. Downstream of the main chamber, a detector chamber can be placed that houses the front pnCCD detector plane (6) that consists of two detector halves which can be moved independently to catch large scattering angles. (7) A second detector plane can be placed at a fixed position for small-angle scattering. The experimental chamber is on a frame that can be translated in the *XYZ* direction as well as pitched and tilted with respect to the FEL beam, which points downwards by 6° after exiting the KB focusing system.

The CAMP instrument consists of several sections along the FEL propagation direction, as can be seen in Fig. 5 ▸. After the KB-mirror chamber, the FEL passes a differential-pumping section that can keep about two orders of magnitude pressure difference to the main chamber and houses a drilled laser mirror for (near-)collinear laser in-coupling. Downstream of this section is the CAMP main chamber, which is placed on a motorized five-axis frame and decoupled from the FEL and laser alignment by a bellow. The foci of the FEL and laser beams are nominally in the center of the CAMP interaction chamber. Around this interaction point, there are four DN250CF flanges (each at 260 mm distance) that can be utilized by the users according to their experimental needs, *i.e.* to mount various combinations of detectors and sample environments. For example, the interaction chamber can be equipped with an ion-electron spectrometer (described in §3.2) as well as various target injection systems that have been developed or adapted for CAMP in the course of experiments at FLASH and LCLS within collaborations (Chapman *et al.*, 2011 ▸; Seibert *et al.*, 2011 ▸; Rajkovic *et al.*, 2010 ▸; Rudek *et al.*, 2012 ▸; Gorkhover *et al.*, 2012 ▸; Loh *et al.*, 2012 ▸; Hau-Riege *et al.*, 2012 ▸; Erk *et al.*, 2013*a* ▸, 2014 ▸; Küpper *et al.*, 2013 ▸; Gomez *et al.*, 2014 ▸; Boll *et al.*, 2014 ▸; Rolles *et al.*, 2014 ▸).

An additional chamber, downstream of the interaction chamber, can house up to two sets of pnCCD detectors placed after the focus to detect forward-scattered photons. This photon-detector system will be discussed in more detail in §3.1. Both pnCCD detectors can be removed individually if they are not needed in order to provide more flexibility inside the chamber as well as to achieve better vacuum, *e.g.* for gas-phase ion and electron spectroscopy on dilute targets. The typical operation base pressure is 10^−7^ mbar with the pnCCDs installed, but can be 10^−10^ mbar after bake-out and without pnCCDs.

The CAMP chamber is fully motorized and can be aligned through *XYZ*-motion as well as pitch and tilt, with respect to the FEL axis. The chamber is tilted downwards by 6° with respect to the horizontal and sidewards to the incoming beam due to the KB focusing mirrors being at 3° grazing incidence.

In order to facilitate experiments that combine the FLASH XUV pulses with femtosecond optical laser light, for example in pump–probe experiments, CAMP has a permanently installed laser table next to the experimental chamber. A grating compressor for the 10 Hz laser system that has an output of up to 10 mJ at 60 fs pulse length with a central wavelength of 810 nm (Redlin *et al.*, 2011 ▸) is installed behind the laser transport-beamline. The second and third optical harmonics of the compressed IR-laser pulse, at 405 nm and 270 nm, respectively, are also available to users (Burt *et al.*, 2017 ▸; Rolles *et al.*, 2018 ▸). They are generated *via* second-harmonic and sum-frequency generation using two beta-barium borate (BBO) crystals. The delay between laser and FEL pulses can be controlled *via* a delay-stage in the range up to 10 ns in femtosecond step size. Additionally, the laser table houses beam-stabilization monitors, virtual-focus imaging, an on-line spectrometer and a single-shot autocorrelator. The laser beams can be coupled into the CAMP main chamber, (near-)collinearly with the FEL beam, by means of a drilled mirror inside the differential pumping chamber in between the KB vacuum tank and the main chamber. Pump–probe experiments using the 810 nm fundamental wavelength NIR pulses have shown that an overall temporal resolution of about 110 fs (FWHM) can be achieved using 60 fs (FWHM) laser pulses and 70 fs (FWHM) FEL pulses. Further technical details on these experiments and, in particular, on the appropriate ways for compensating for the temporal jitter between the optical laser and the FEL in the data analysis, are given by Savelyev *et al.* (2017 ▸) and Rolles *et al.* (2018 ▸). Other experiments using the third optical harmonic of the IR laser have achieved an overall temporal resolution between 120 fs and 200 fs (FWHM) with longer UV pulse lengths of about 100–170 fs (FWHM) (Brauße *et al.*, 2018 ▸; Fisher-Levine *et al.*, 2018 ▸; Amini *et al.*, 2018 ▸). A high-repetition-rate burst-mode laser can also be used (Redlin *et al.*, 2011 ▸). The BL1 area has a permanent laser-safety enclosure with a footprint of about 3 m by 4 m that houses the laser table and the CAMP end-station.

## 3. Detector systems: pnCCD photon-detectors and charged particle spectrometers   

The CAMP end-station can provide a suite of electron-ion spectrometers as well as large-area photon detectors that are described in the following. To further illustrate the experimental capabilities of these instruments, some results obtained during the instrument commissioning are shown.

### 3.1. Large-area pnCCD photon-detectors   

The CAMP end-station can be equipped with two large-area pn-junction charge coupled device (pnCCD) detectors (Hartmann *et al.*, 2008 ▸; Reich *et al.*, 2008 ▸; Strüder *et al.*, 2010 ▸). Each detector plane has a sensitive area of 77 mm by 77 mm, with a total of 1 Mpixel (75 µm × 75 µm pixel size), and has single-photon-counting capabilities. The pnCCDs collect scattered and fluorescence photons with high quantum efficiency and an energy resolution of 40 to 200 eV between <100 eV and 25 keV at full-frame readout rate of up to 200 Hz. Due to the low capacitance of the readout anode combined with column-parallel readout, the pnCCD detector offers a very good signal-to-noise ratio. This enables single photon detection and counting even for low-energy photons of 50 eV and above. The pnCCD can be operated in different gain modes, *i.e.* from highest to lowest, full-, 1/4, 1/16, 1/64 or 1/256-gain. In the case of 90 eV photons, these correspond to a maximum number of photons per pixel of 125, 500, 2000, 8000 and 32000, respectively.

One detector system is composed of two modules (lower and upper halves). A variable-sized gap between the upper and lower module of the segmented front pnCCD detector plane (each module is 76.8 mm wide and 38.4 mm high) allows the direct FEL beam (and, in some experiments, a high-power optical pump–probe laser) to pass through the detector, while the small-angle scattering signal within the area of the gap can be detected on the second pnCCD, which has a fixed-diameter hole for the direct FEL beam to pass through and exit the CAMP end-station. The movable front pnCCD detector can cover scattering angles from ∼3° to 28° (around the FEL-beam) in the position closest to the focus (70 mm) and closest to the beam axis (*i.e.* the gap between the two modules is fully closed), while covering angles up to ∼50° when being moved further away from the beam axis (*i.e.* maximal gap between two modules). The front detector plane can also be moved along the beam propagation direction from 70 mm up to 220 mm away from the focus spot (the lower pnCCD module is thereby at 2 mm larger distance compared with the upper module).

**Figure 6:**
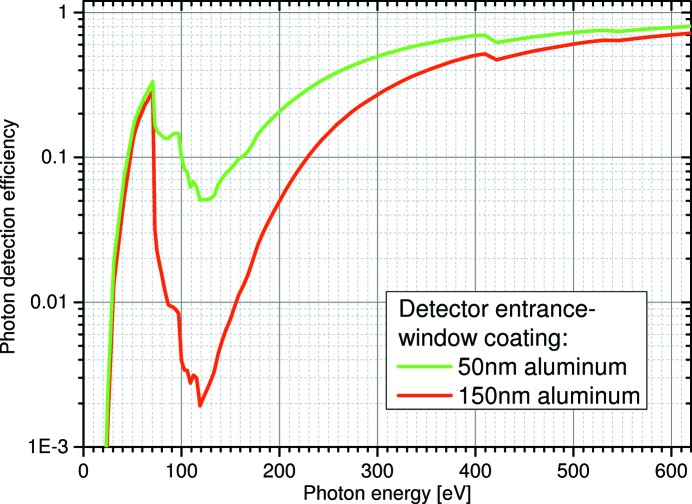
Photon detection efficiency of the pnCCD detectors in the energy range below 600 eV. The red and green curves show the quantum efficiency of the detector modules with 50 nm and 150 nm aluminium entrance windows, respectively. These coatings were introduced to suppress detector background resulting from optical light, *e.g.* from stray light of strong optical lasers or ambient light inside the experimental chamber from windows or other instrumentation.

In order to suppress the background signal created by optical light inside the chamber, *e.g.* from entering ambient light or a strong IR-pump laser, the pnCCD modules have coated entrance windows of either 50 nm- or 150 nm-thick aluminium layers. Stray light inside the chamber from high-power lasers, with up to several mJ of power, can be significant, and the aluminium coatings are not always sufficient to fully suppress the optical laser light. The resulting increased background by the optical laser can make the clean detection of small signal levels at small angles challenging. However, proper stray light control by apertures and collimating tubes, combined with the option to subtract the optical laser background from the XUV photon signal, can provide scattering images also for pump–probe experiments that employ a strong optical laser. The aluminium coatings also affect the photon detection efficiency at XUV energies, especially just above the aluminium (2*p*)-absorption edge (>70 eV) where the efficiency drops by a factor of >30 for the modules coated with 150 nm aluminium. Fig. 6 ▸ shows the calculated quantum efficiency of the pnCCD detectors for the energy range up to 600 eV (Strüder *et al.*, 2010 ▸).

**Figure 7:**
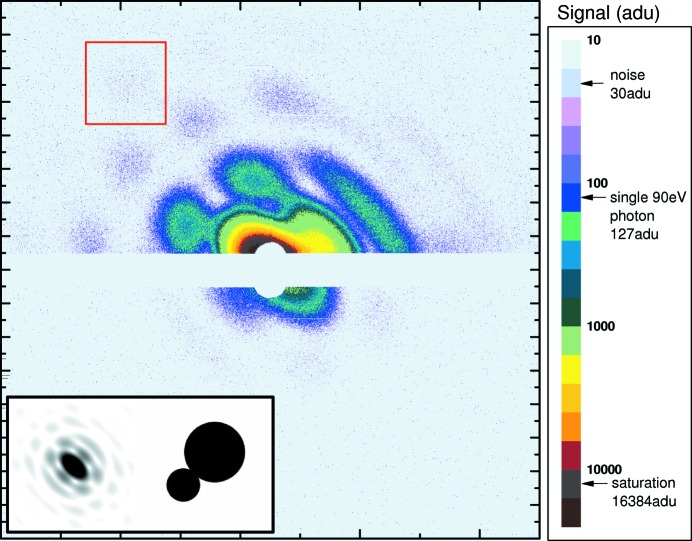
Scattering pattern of a single, large, non-spherical xenon twin-cluster obtained with a single FEL pulse of 90 eV photon energy (60 µJ, 100 fs pulses). A maximum transferred momentum of 0.3 nm^−1^ could be obtained within this experiment. The combination of different pnCCD modules with 50 nm and 150 nm Al coating used in this experiment allowed for an even larger dynamic range (see color scale on the right side). The image on the upper module (50 nm Al coating) shows both saturation in the center of the detector as well as single photon signal, *e.g.* in the area indicated by the red box. On the lower detector module (150 nm Al coating) the unsaturated scattering signal in the zero-order maximum could be retrieved simultaneously. The inset on the lower left side shows a simulated image (two-dimensional FFT of the given outline). The non-spherical geometry of the nano-cluster is a signature of the cluster growth by coagulation (Rupp *et al.*, 2012 ▸), a gas-phase process that can be studied only *via* single-particle diffractive imaging.

An example of the imaging capabilities of the CAMP end-station is given in Fig. 7 ▸, where a xenon twin-cluster is imaged in-flight by a single FEL pulse at 90 eV photon energy. This scattering pattern was recorded with the pnCCD in the highest gain mode.

Within the detector geometry (70 mm distance to the focus, 4 mm gap between the two modules) used for this image, a maximum scattering angle of 38° and a corresponding maximum transferred momentum of *q* = 0.3 nm^−1^ was obtained. Values up to 0.8 nm^−1^ have been obtained using shorter wavelengths. The signal in the lower part of the upper module is saturated, corresponding to more than 130, 90 eV photons per pixel. For the measurement, detector modules with different aluminium entrance windows were used, resulting in a factor of 15 lower detection efficiency for the scattered 90 eV photons on the lower module. This helped facilitate the retrieval of an unsaturated signal of the zero-order maximum of the scattering pattern.

### 3.2. Electron and ion spectrometers   

**Figure 8:**
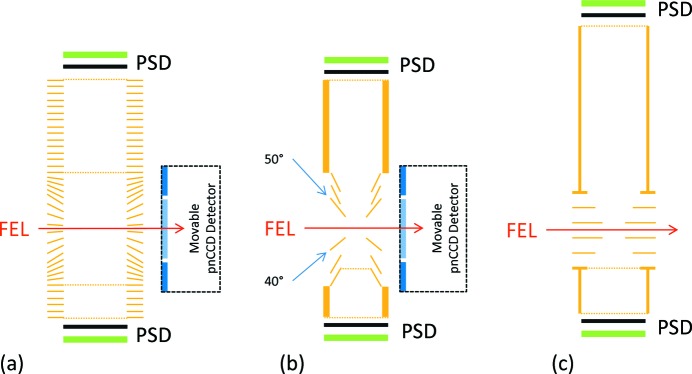
Schematic of the three double-sided electron-ion spectrometers for the CAMP@FLASH end-station. (*a*) Reaction microscope for coincidence measurements of electrons and ions and ion-momentum spectroscopy; (*b*) double-sided conical-electrode VMI spectrometer for simultaneous, ‘shadow-less’ operation with pnCCD scattering detectors; (*c*) double-sided flat-electrode VMI spectrometer with improved energy resolution. All spectrometers can be equipped with delay-line anodes or phosphor screens as position-sensitive detectors (PSDs).

Three double-sided charged-particle imaging spectrometers are available for the CAMP instrument, see Fig. 8 ▸, designed to detect ions and electrons of up to several hundred electronvolts kinetic energy simultaneously, within the full 4π solid angle. They can be operated in different modes for time-of-flight mass spectrometry, velocity map imaging (VMI) (Eppink & Parker, 1997 ▸) or recoil ion momentum spectroscopy with a REaction MIcroscope (REMI) (Ullrich *et al.*, 2003 ▸).

The spectrometers can all be equipped with two position-sensitive detectors at top and bottom. These consist of two 80 mm MCP detectors in chevron configuration, combined with either delay-line anodes (RoentDek DLD80 on the top side and RoentDek HEX80 on the bottom side) or phosphor screens (Photonis APD 2 PS 75/32/25/8 I 60:1); typically a P20 phosphor screen is used on the bottom/electron side and a fast P47 phosphor screen is used on the top/ion side of the spectrometer. The phosphor screens are imaged at 10 Hz by CCD cameras (Allied Vision Pike F-145B) outside of vacuum. Delay-line anodes are suitable for coincident momentum-resolved few-particle detection, in particular in the burst-mode operation of FLASH, while phosphor screens allow for high count rates of several hundred or more charged particles per frame in the 10 Hz ‘single bunch’ FEL mode.

Each spectrometer is intended for different applications: the REMI spectrometer, see Fig. 8(*a*) ▸, in combination with delay-line anodes is ideally suited to measure the time-of-flight and hit positions of ions extracted from a narrow focal region in order to reconstruct their three-dimensional momentum with a few meV resolution. A long (top) and a short (bottom) side are available, which are appropriate for ion kinetic energies of <100 eV and <200 eV per charge, respectively. This allows for 3D-momentum-spectroscopy of highly charged high-energy ions detected in coincidence, for example produced in Coulomb explosion of molecular systems created in a high-intensity FEL focus.

The main purpose of the VMI spectrometers, typically equipped with two phosphor screens, is to record angle-resolved electron and ion spectra simultaneously for an extended interaction region of up to several millimeters and for high particle count rates per FEL shot.

The asymmetrically shaped electrodes of the conical-electrode VMI (CE-VMI, see Fig. 8*b* ▸) are intended for detection of scattered photons simultaneous to ions and electrons, and are designed in a way that the shadow projected onto the pnCCD sensor at large scattering angles is minimized. Due to the open electrode geometry around the interaction region, the extraction fields are, in terms of potential curve confinement and homogeneity, not as well defined as for a typical flat-electrode single-sided VMI spectrometer (Eppink & Parker, 1997 ▸). Therefore, focusing capabilities are very sensitive to the exact vertical position of the interaction region with respect to the two central electrodes, reducing the overall energy resolution. The CE-VMI spectrometer can collect electrons in a 4π solid angle for up to 250 eV kinetic energy on the bottom side, and has a demonstrated energy resolution of ∼1 eV for 50 eV kinetic energy (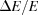 = 2%) (Brauße *et al.*, 2018 ▸).

On the other hand, the flat-electrode VMI spectrometer (FE-VMI, see Fig. 8*c* ▸) (Bomme *et al.*, 2018 ▸) was designed without considering constraints for simultaneous use of large-angle imaging detectors and has a 4π acceptance for electrons with up to 300 eV energy on the bottom side. Simultaneously, ions with kinetic energies up to 40 eV, for singly charged ions, can be measured on the top side. The energy resolution for electrons was shown to be better than 4.5 eV for 300 eV (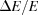 < 1.5%) photo-electrons and better than 400 meV for 5 eV carbon ions (Bomme *et al.*, 2018 ▸).

Recently, we have demonstrated that both VMI spectrometers can be combined with novel time-stamping cameras employing fast imaging sensors (Amini *et al.*, 2017 ▸; Brauße *et al.*, 2018 ▸; Burt *et al.*, 2017 ▸; Fisher-Levine *et al.*, 2018 ▸). The Pixel Imaging Mass Spectrometry (PImMS) camera (John *et al.*, 2012 ▸; Amini *et al.*, 2015 ▸) and the TimepixCam (Fisher-Levine & Nomerotski, 2016 ▸) allow for simultaneous detection of a high-resolution ion mass spectrum as well as two-dimensional momenta of all ionic fragments with a temporal resolution of a few tens of nanoseconds, thus making high-voltage gating of the detector to detect only one specific fragment ion obsolete. These camera systems are currently only available through collaborations.

## 4. Summary and outlook   

In summary, CAMP@FLASH at beamline BL1 is a multi-purpose end-station tailored for diffraction-imaging of nano-structures and magnetic domains as well as for electron- and ion spectroscopy, often performed in a pump–probe scheme using ultra-short FEL pulses in combination with the optical femtosecond pump–probe laser available at FLASH1.

The wavelength-independent tight focusing of the KB mirrors in combination with the pnCCD imaging detectors enables scattering measurements, *e.g.* of single nanoparticles, across the full parameter range of FLASH1. First experiments include both measurements of static processes, such as magnetic scattering from magnetic domains and single particles, as well as optical-laser-pump FEL-probe experiments, investigating femtosecond dynamics in thin-films and single particles, with sizes from a few micrometers down to 10 nm in diameter. Other experiments utilize the double-sided VMI spectrometers, in combination with the permanent laser setup and the FEL pulses, to investigate femtosecond molecular dynamics in the gas phase, for example by means of Coulomb explosion imaging, ion-electron correlation spectroscopy or ion-covariance mapping.

The system is ready for future upgrades of the FLASH accelerator towards higher photon energies and for operation with higher-order harmonics, as the beamline mirrors and detector systems are fully capable of going to the oxygen *K*-edge photon energy and beyond.

In order to allow for XUV–XUV pump–probe experiments, a multilayer-mirror-based split-and-delay unit, called DElay-Stage for CAMP (DESC) (Sauppe *et al.*, 2018 ▸), can be inserted to split the FEL pulses, delay these two beams by −29 ps to 654 ps with 200 as (mechanical) accuracy, and overlap them in the focus of the KB mirror system.

Combining large-area pnCCD photon-detectors and large-acceptance ion- and electron-coincidence spectrometers with a tight wavelength-independent KB focusing, the CAMP end-station provides new possibilities for users at FLASH to study a wide range of processes in samples from single atoms in the gas phase to large nanoparticles and solid-state samples using single FEL pulses, fs-laser/FEL pump–probe or FEL/FEL pump–probe methods.

Information on the CAMP@FLASH end-station, the BL1 beamline, as well as on user access to this experimental station can be found on the FLASH (DESY, 2017*a* ▸,*c* ▸) and DESY Photon Science (DESY, 2017*b* ▸) websites.
